# Effectiveness and safety of sumatriptan 85 mg/naproxen sodium 500 mg combination for acute migraine: prospective real-world evidence from the GRASP Study Group

**DOI:** 10.3389/fneur.2026.1912200

**Published:** 2026-07-29

**Authors:** Dimitrios Rikos, Andreas A. Argyriou, Emmanouil V. Dermitzakis, Panagiotis Soldatos, Nikolaos Dimisianos, Christos Tsironis, Eleni Mavraki, Maria Chondrogianni, Aikaterini Foska, Evangelia Dimitriadou, Emmanouil Giakoumakis, Michail Vikelis, on behalf of Greek Research Alliance for the Study of Headache and Pain (GRASP) Study Group

**Affiliations:** 1 404 Military Hospital, Larisa, Greece; 2 Department of Neurology, Headache Outpatient Clinic, Agios Andreas General Hospital of Patras, Patras, Greece; 3 General Clinic, Thessaloniki, Greece; 4 Kalamata Headache Clinic, Kalamata, Greece; 5 General Neurology Clinic, Corfu, Greece; 6 Department of Neurology, Headache Outpatient Clinic, University Hospital of Ioannina, Ioannina, Greece; 7 Department of Neurology, Headache Outpatient Clinic, University General Hospital of Alexandroupolis, Alexandroupolis, Greece; 8 Second Department of Neurology, National and Kapodistrian University of Athens, School of Medicine, “Attikon” University Hospital, Athens, Greece; 9 NeuroCrete Headache Clinic, Heraklion, Greece; 10 Glyfada Headache Clinic, Glyfada, Greece

**Keywords:** acute migraine, acute treatment, combination of sumatriptan and naproxen sodium, effectiveness, safety

## Abstract

**Objective:**

This prospective multicenter observational study, designed by the Greek Research Alliance for the Study of Headache and Pain (GRASP), evaluated the real-world effectiveness and tolerability of oral fixed-dose sumatriptan 85 mg/naproxen sodium 500 mg across repeated migraine attacks.

**Methods:**

Adults with episodic or chronic migraine for whom the treating physician had selected the oral fixed-dose combination as acute treatment were enrolled. Outcomes were recorded for up to three consecutive treated migraine attacks. The primary endpoint was 2-h pain freedom. Secondary endpoints included 2-h pain relief, sustained pain freedom at 24 h without rescue medication, return to normal activities, resolution of associated symptoms, treatment-timing effects, subgroup outcomes, and adverse events.

**Results:**

Overall, 111 patients contributed 320 treated attacks. Two-hour pain freedom was achieved in 76/320 attacks (23.8%; 95%CI: 19.4–28.7), 2-h pain relief in 220/320 attacks (68.8%; 95%CI: 63.5–73.6), full return to normal activities in 206/320 attacks (64.4%; 95%CI: 59.0–69.4), and no rescue medication use in 246/320 attacks (76.9%; 95%CI: 72.0–81.2). Associated symptoms improved substantially, with 2-h resolution of photophobia, phonophobia, and nausea/vomiting occurring in 49.0, 51.0, and 63.7% of affected attacks, respectively. Early treatment within 1 h was associated with higher pain relief and functional recovery. Adverse events, occurring in 20.0% of attacks, were mainly gastrointestinal symptoms, dizziness, chest pressure, and somnolence/fatigue.

**Conclusion:**

Fixed-dose sumatriptan/naproxen sodium was associated with modest 2-h pain freedom but clinically meaningful pain relief, functional recovery, and low rescue medication use. Tolerability was consistent with the known profiles of triptans and non-steroidal anti-inflammatory drugs.

## Highlights

- Fixed-dose sumatriptan/naproxen sodium achieved 2-h pain relief in 68.8% and 2-h pain freedom in 23.8% of 320 treated migraine attacks.
- Full return to normal activities occurred in 64.4% of attacks by 2 h.
- Rescue medication was avoided in 76.9% of treated attacks, supporting durable attack control in routine practice.
- Patients with episodic migraine showed significantly better outcomes than those with chronic migraine across pain freedom, sustained pain freedom, function, and rescue medication use.
- Early treatment within 1 h was associated with significantly higher pain relief and functional recovery.

## Introduction

Migraine affects more than one billion people worldwide and consistently ranks among the leading causes of years lived with disability (1). Recent Global Burden of Disease analyses confirm this burden and underscore that better acute treatment is a clinically important public health priority (2). Migraine attacks are clinically heterogeneous, typically characterized by unilateral moderate/severe throbbing pain or a pulsating sensation lasting 4–72 h that is accompanied by at least one associated symptom, including nausea, vomiting, and extreme sensitivity to light or noise. The therapeutic goal is, therefore, to achieve a rapid and sustained restoration of function, with relief of pain and associated symptoms, minimal recurrence, little need for rescue medication, and a manageable side-effect profile (3).

In current routine clinical practice, oral formulations of triptans, including sumatriptan, almotriptan, eletriptan, frovatriptan, naratriptan, rizatriptan, and zolmitriptan, as well as non-steroidal anti-inflammatory drugs (NSAIDs) are the most widely used options for treating acute migraine, while newer gepants and ditans have further widened the therapeutic arsenal, especially for patients with vascular contraindications or poor tolerability (3, 4). Recent comparative evidence suggests that triptans remain among the most effective oral acute treatments for obtaining 2-h pain freedom, reinforcing their continued clinical relevance despite the entry of newer drug classes (5). At the same time, incomplete response, attack recurrence, adverse effects, and treatment inconsistency remain common in everyday practice of acute migraine treatment with triptans, which is precisely why real-world data remain informative and clinically important (6).

The fixed-dose combination of sumatriptan and naproxen sodium within a single tablet was developed to address different biological components of a migraine attack. Sumatriptan, a 5-HT1B/1D receptor agonist, modulates trigeminovascular activation and migraine-specific nociceptive transmission, whereas naproxen inhibits cyclo-oxygenase activity and prostaglandin-mediated inflammatory mechanisms that may contribute to sustained peripheral and central sensitization (7, 8). This dual mechanism justifies combining a migraine-specific agent with a longer-acting anti-inflammatory drug, particularly when the therapeutic goal is not only early pain reduction but also a durable response without recurrence or intake of additional rescue medication (9–11).

Available randomized trials and subsequent reviews have shown that sumatriptan/naproxen sodium is superior to placebo and, in several analyses, to its individual components, i.e., sumatriptan or naproxen given as monotherapy, for clinically relevant acute endpoints, including two-hour pain freedom and sustained pain-free response (7–13). Additional studies have supported its effectiveness in early treatment, probable migraine, menstrual migraine, and patients with functional impairment, while patient-reported outcomes have suggested gains in productivity, satisfaction, and return to daily living activities (14–18). Safety observations have generally been consistent with the known profiles of triptans and NSAIDs, although gastrointestinal symptoms, dizziness, somnolence, chest pressure, blood pressure concerns, and individual cardiovascular risk assessment remain clinically relevant (19).

However, the results of controlled trials do not fully translate into everyday treatment use in specialist headache practice, where patients have variable migraine frequency, long disease duration, chronic migraine, prior medication exposure, psychiatric and medical comorbidities, delayed intake, and heterogeneous attack characteristics. Real-world data from Greek headache practice are limited. The present study therefore prospectively evaluated the real-world effectiveness and safety of the oral fixed-dose sumatriptan 85 mg/naproxen sodium 500 mg combination for up to three consecutive migraine attacks under routine clinical conditions. Our primary hypothesis was that this combination would provide clinically meaningful pain relief, functional recovery, and a low need for rescue medication in everyday headache practice.

## Patients and methods

### Study design and setting

This was a multicenter, prospective, observational real-world study conducted by the GRASP Study Group in Greek adults receiving fixed-dose sumatriptan 85 mg/naproxen sodium 500 mg for the acute treatment of migraine. The study was designed to reflect routine clinical use in 11 participating headache centers from both public and private settings, covering 10 Greek major urban geographic areas. No randomization, blinding, or control group was used.

### Participants

Without performing an apriori formal power analysis, the research was conducted from October 2025 to February 2026. This was an observational, real-world registry study rather than a randomized controlled trial. Therefore, the sample size was determined by the number of consecutive eligible patients who presented to the participating clinics and were prescribed the medication during the predefined study period, rather than by a formal statistical power calculation.

In line with the predefined inclusion criteria, we recruited consecutive adults with either episodic (EM) or chronic migraine (CM) for whom the treating physician had selected to commence the fixed-dose combination fixed-dose sumatriptan 85 mg/naproxen sodium 500 mg (Suvexx^®^, F. C. Tab [85 + 500] Mg/Tab Bt X 9 Tabs, Orion Pharma) as acute oral therapy. Sumatriptan 85 mg/naproxen sodium 500 mg was given, according to standard clinical practice, as a symptomatic treatment of a migraine attack (with or without aura) of any severity (mild, moderate or severe headache pain intensity). The use of antiemetics was permitted according to the treating physician’s standard clinical practice. Patients were allowed to use rescue medications of their or their physician’s choice if pain relief was inadequate after 2 h. The most frequently utilized rescue medications were simple analgesics (i.e., paracetamol), other NSAIDs (i.e., ibuprofen, diclofenac), or an additional dose of a triptan, provided it did not exceed the maximum daily recommended dose.

Patients with a known history of major cardiovascular disease, uncontrolled hypertension, stroke/TIA history, peripheral vascular disease, severe hepatic or renal disease, peptic ulcer disease, anticoagulant use, known hypersensitivity, and Gastroesophageal reflux disease (GERD), were excluded. All patients with psychiatric comorbidity had mild-to-moderate, clinically stable conditions (predominantly anxiety and depression) managed in an outpatient setting, with no cognitive impairment or acute exacerbations that would compromise decision-making capacity or the reliability of symptom self-reporting.

Migraine was diagnosed in accordance with the 2018 International Classification of Headache Disorders-third edition, criteria (20). Eligibility was confirmed using a protocol-specific checklist. All participants provided written informed consent, and the study was approved by the Institutional Review Board of Agios Andreas State General Hospital of Patras (protocol number 7–2/7/2025), in accordance with the principles of the Declaration of Helsinki. The study was not registered in a public clinical trial registry, as it forms part of the ongoing migraine registry maintained by the GRASP Study Group. This registry has not been assigned a formal trial or registry identifier in either a public or internal repository, while it is conducted under the approval of the relevant institutional ethics committees and oversight by the GRASP steering committee.

### Data collection

Baseline demographic and clinical data for enrolled patients were collected, including age, gender, body mass index, migraine frequency category, relevant comorbidities, and previous acute migraine medication exposure. Per-attack records for up to three consecutive treated attacks for each individual patient, including pain intensity, associated symptoms (nausea, phonophobia, photophobia), return to function, rescue medication use and adverse events, were also recorded.

For each treated attack, pain intensity was documented at baseline, 2 h, and 24 h after intake using an 11-point numeric rating scale (NRS), where 0 represented no pain and 10 represented the worst pain. To facilitate interpretation, the NRS was mapped to a four-point clinical scale: 0, no pain; 1–3, mild pain; 4–6, moderate pain; and 7–10, severe pain, according to International Headache Society (IHS) consensus guidelines (21). Associated symptoms, including nausea or vomiting, photophobia (aversion to light), and phonophobia (aversion to noise), were graded on a four-level categorical scale as absent, mild, moderate, or severe.

### Outcomes

The primary endpoint was pain freedom at 2 h, defined as no pain (NRS = 0) at 2 h while the pain relief secondary endpoint was defined as improvement from moderate or severe baseline pain (NRS ≥ 4) to mild or no pain (NRS ≤ 3) at 2 h, consistent with IHS acute trial principles (21). Sustained pain freedom was defined as pain freedom at both two and 24 h without rescue medication. Full return to normal activities at 2 h was recorded only when the response was explicitly “yes”; partial return was treated as non-response to preserve a conservative functional endpoint. Resolution of associated symptoms was defined as complete absence of the symptom at 2 h among attacks in which that symptom was present at baseline.

Patient satisfaction was assessed per attack on a 4-point ordinal scale (0 = Not at all, 1 = A little, 2 = Much, 3 = Very much) and analyzed as both a continuous ordinal variable and dichotomized binary endpoints. The primary binary endpoint was “Satisfied” (score ≥ 2); a secondary endpoint was “Highly Satisfied” (score = 3).

The attacks treated were stratified by attack number, i.e., 1st; 2nd and 3rd, to evaluate consistency of response. Attack-level outcomes were described overall and by consecutive attack number to explore response consistency during repeated use. Patient-level consistency was summarized by the number of treated attacks in which two-hour pain freedom was achieved. Subgroup comparisons between EM and CM, and between patients with and without psychiatric comorbidity, were restricted to the first recorded attack for each patient (*n* = 111) to preserve independence of observations. The effect of treatment timing was evaluated at attack level by comparing early intake, defined as treatment within 1 h from symptom onset, with later intake after the first hour. The comparison between these two groups was conducted at the attack level (*n* = 320).

Adverse events were systematically recorded for each treated attack at 2 and 24 h post-treatment as binary outcomes, accompanied by an open-text field describing the event and were categorized *post-hoc* into clinically homogeneous groupings.

### Statistical analysis

Categorical variables are presented as counts and percentages. Continuous variables are presented as mean ± standard deviation and, where available, median and range. Ninety-five percent confidence intervals (95%CIs) for key proportions were calculated using the Wilson method. Subgroup comparisons between EM and CM and between patients with and without psychiatric comorbidity were restricted to the first recorded attack for each patient (*n* = 111) to preserve independence of observations. Bivariate associations with effectiveness outcomes (pain freedom, pain relief ≥50%, sustained pain freedom, full return to activities at 2 h, absence of rescue medication) were tested using chi-square or Fisher’s exact test for proportions and Mann–Whitney U for the ordinal score; correlations were assessed via Spearman’s r. Categorical variables were compared using Pearson’s chi-square test, with Fisher’s exact test reported when expected cell counts were below five. Statistical significance was set at *p* < 0.05. Repeated-attack outcomes were described overall and by attack number. Because attacks from the same patient are correlated, repeated-attack comparisons should be interpreted descriptively. No formal mixed-effects or generalized estimating equation model was applied. All estimates at the attack level should be treated as descriptive since the analysis did not formally account for clustering of attacks within individual patient. The early-versus-late treatment comparison was performed at the attack level (*n* = 320) and should be interpreted as associative rather than causal because it was unadjusted for within-patient correlation and other confounders. Analyses were performed using IBM SPSS Statistics, release 31.0 (SPSS Inc., Chicago, IL, United States).

## Results

A total of 111 patients were analyzed, of whom 102 (91.9%) contributed three consecutive treated attacks, resulting in a total of 320 migraine attacks separately treated with the fixed-dose combination sumatriptan 85 mg/naproxen sodium 500 mg. The most commonly used antiemetics were metoclopramide and domperidone.

The mean age of our study cohort was 42 ± 11 years and 80.2% were women. Mean baseline pain intensity at the first treated attack was 7.3 ± 1.7 on the 0–10 NRS. Attacks with moderate pain intensity (NRS ≥ 4) at baseline were 112/320 (35%) and with severe intensity 208/320 (65%). Detailed baseline characteristics are presented in Table 1.

**Table 1:** Baseline demographic and clinical characteristics of our study cohort, comprised by a total number of 111 participants.

Variable at baseline	N/mean ± SD	% / median (range)
Age, years	42.04 ± 10.95	44 (19–65)
Female gender	89	80.2%
BMI, kg/ m^2^	24.39 ± 3.47	24.4 (17.3–33.1)
Baseline pain intensity (first attack, NRS 0–10)	7.26 ± 1.74	8 (4–10)
Migraine frequency		
1–7 days/month	46	41.4%
8–14 days/month	32	28.8%
>15 days/month	33	29.7%
Acute medication history (any-time use)		
Rizatriptan	88	79.3%
Sumatriptan	63	56.8%
Eletriptan	47	42.3%
Frovatriptan	17	15.3%
Comorbidities		
Psychiatric	28	25.2%
Endocrine/metabolic	24	21.6%
Minor well-controlled cardiovascular	16	14.4%
Autoimmune	6	5.4%

Two-hour pain freedom was achieved in 76/320 treated attacks (23.8%; 95%CI: 19.4–28.7), while 2-h pain relief was achieved in 220/320 attacks (68.8%; 95%CI: 63.5–73.6). Sustained pain freedom was achieved in 70/320 attacks (21.9%; 95%CI: 17.7–26.7), with rates of 22.5% (25/111), 19.6% (21/107) and 23.5% (24/102) across the first, second and third attacks, respectively. Only a small proportion of patients achieving pain freedom at 2 h did not achieve sustained pain freedom in 24 h (6/76; 7.9%). Mean baseline NRS pain intensity dropped significantly from 7.3 ± 1.7 to 3.1 ± 2.6 at 2 h after the first treated attack (*p* < 0.001). Rescue medication was required in 74/320 attacks overall (23.1%). Full return to normal activities at 2 h occurred in 206/320 attacks (64.4%; 95%CI: 59.0–69.4), and rescue medication was avoided in 246/320 attacks (76.9%; 95%CI: 72.0–81.2).

**Figure 1:**
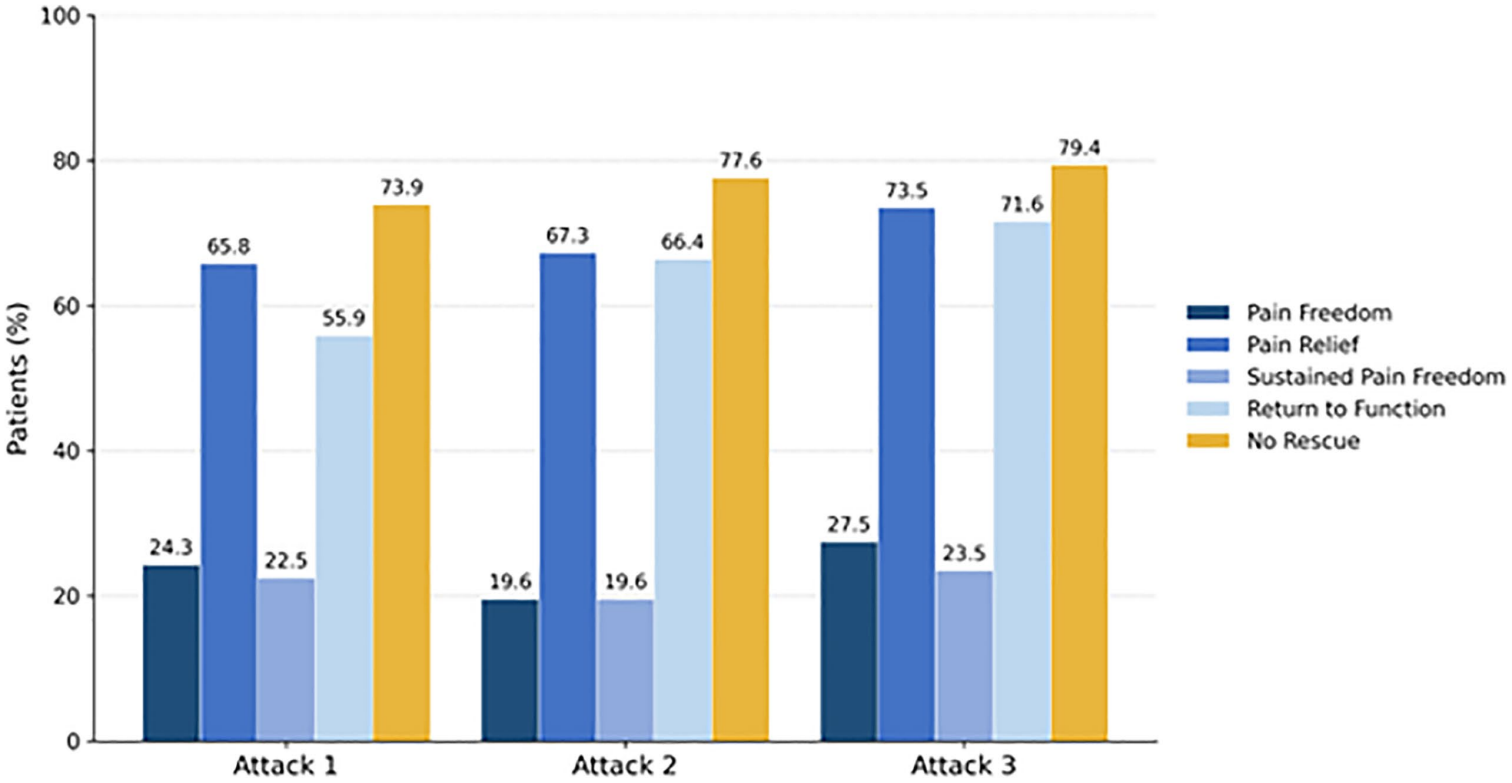
Effectiveness outcomes across consecutive treated attacks.

Rates across consecutive attacks were 55.9% (62/111), 66.4% (71/107) and 71.6% (73/102) for the first, second and third attack, respectively, showing numerically higher functional recovery across recorded attacks. Effectiveness outcomes overall and across consecutive treated attacks are shown in Table 2 and Figure 1.

**Table 2:** Effectiveness outcomes overall and across consecutive treated attacks.

Outcome	Overall (*n* = 320)	Attack 1 (*n* = 111)	Attack 2 (*n* = 107)	Attack 3 (*n* = 102)
Pain freedom (2 h)	23.8%	24.3%	19.6%	27.5%
Pain relief (2 h)	68.8%	65.8%	67.3%	73.5%
Sustained pain freedom (24 h)	21.9%	22.5%	19.6%	23.5%
Return to function (2 h)	64.4%	55.9%	66.4%	71.6%
No rescue medication	76.9%	73.9%	77.6%	79.4%

Consistency analysis showed that 40/111 patients (36%) achieved pain freedom in at least one treated attack; 22/111 (19.8%) in at least two attacks and 14/111 (12.6%) achieved pain freedom in all three consecutive treated attacks. Responders (≥1 attack with pain freedom; *n* = 40) showed several baseline characteristics differing from non-responders (*n* = 71). Responders were on average younger (39.8 ± 12.3 vs. 43.3 ± 10.0 years), had lower baseline pain (6.5 ± 2.0 vs. 7.7 ± 1.5), shorter disease duration (18.2 ± 12.2 vs. 19.9 ± 10.9 years) and a lower proportion of women (70.0% vs. 85.9%); BMI was comparable (24.2 ± 3.6 vs. 24.5 ± 3.4 kg/m^2^). By response profile classification, 71/111 patients (64.0%) were never responders, 26/111 (23.4%) inconsistent responders, and 14/111 (12.6%) consistent responders (pain freedom in all three treated attacks).

**Figure 2:**
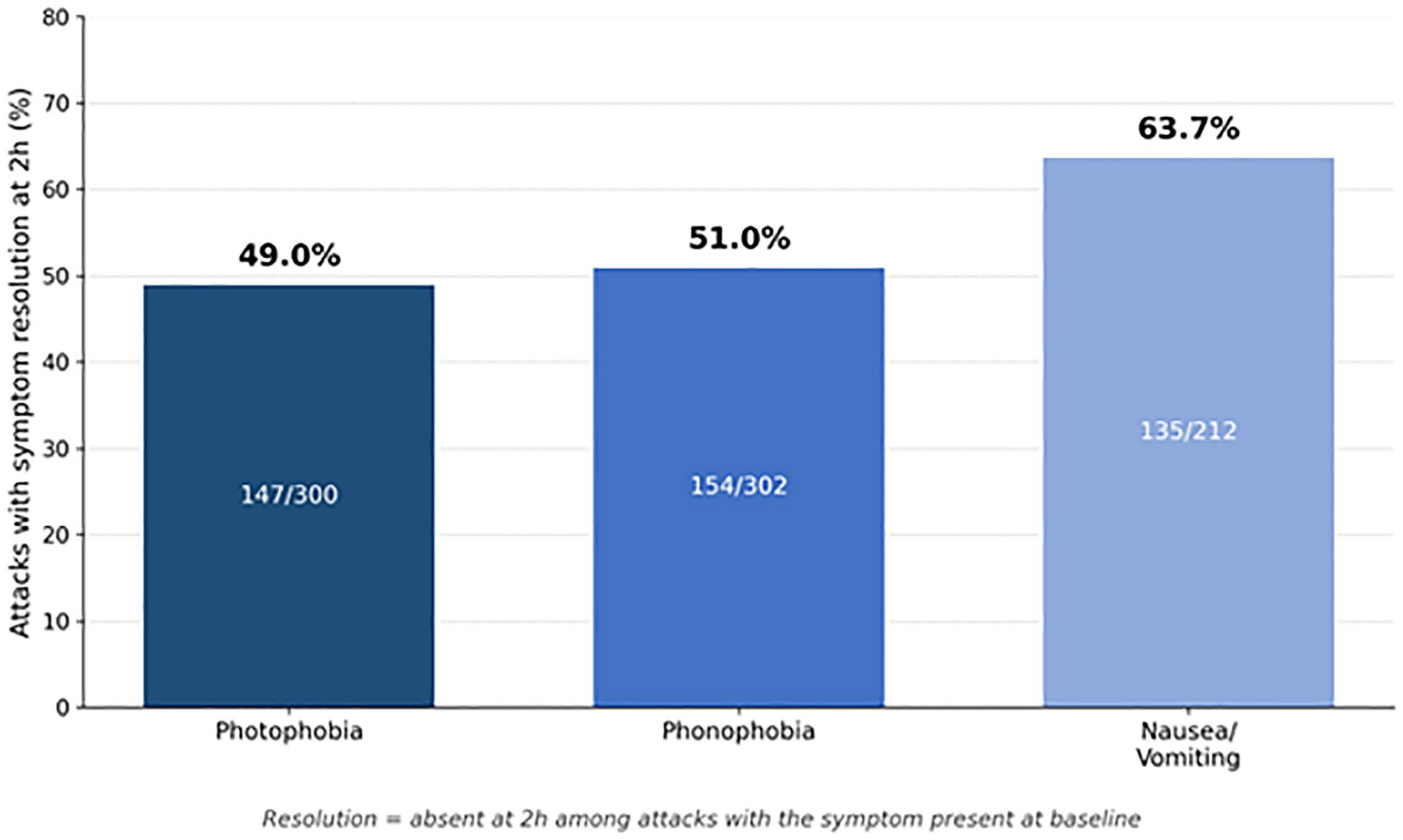
Resolution of associated migraine symptoms at 2 h.

As shown in Figure 2, among attacks with any associated migraine symptom (photophobia, phonophobia, nausea/vomiting) of any severity, complete 2-h symptom resolution was achieved in 147/300 attacks with photophobia (49.0%; 95%CI: 43.4–54.6), 154/302 attacks with phonophobia (51.0%; 95%CI: 45.4–56.6), and 135/212 attacks with nausea or vomiting (63.7%; 95%CI: 57.0–69.9).

Of 111 patients, 78 (70.3%) had EM and 33 (29.7%) CM. Patients with EM demonstrated higher response rates than those with CM across all endpoints. Pain freedom at 2 h was achieved in 30.8% (24/78) of episodic patients versus 9.1% (3/33) of chronic patients (chi-square *p* = 0.015; Fisher’s exact *p* = 0.016). Sustained pain freedom was 28.2% (22/78) vs. 9.1% (3/33), *p* = 0.028; full return to function 65.4% (51/78) vs. 33.3% (11/33), *p* = 0.002; and no rescue medication 79.5% (62/78) vs. 60.6% (20/33), *p* = 0.039. Pain relief was higher in episodic patients but did not reach statistical significance (70.5% vs. 54.5%, *p* = 0.105). Conversely, no statistically significant differences were observed across endpoints between patients with and without psychiatric comorbidity. Pain freedom rates were similar (21.4% vs. 25.3%, *p* = 0.68), while comparable patterns were also observed for pain relief (60.7% vs. 67.5%, *p* = 0.51), sustained pain freedom (21.4% vs. 22.9%, *p* = 0.87) and return to function (53.6% vs. 56.6%, *p* = 0.78).

Regarding the effect of the early treatment of a migraine attack, 236/320 attacks (73.8%) were treated early (≤1 h from symptoms onset) and 84 (26.2%) late. Early treatment was associated with significantly higher rates of pain relief (74.6% vs. 52.4%, *p* < 0.001) and full return to function (71.2% vs. 45.2%, *p* < 0.001). Pain freedom (25.8% vs. 17.9%, *p* = 0.18) and sustained pain freedom (23.7% vs. 16.7%, *p* = 0.22) showed numerical advantages favoring early treatment without reaching statistical significance.

Regarding the patient-reported outcome (PRO) of patient satisfaction, 247 of 320 treated attacks (77.2%) resulted in satisfaction (much or very much), with 48.1% rated in the highest category (very much). Patient satisfaction was strongly and consistently associated with pain freedom at 2 h, yielding satisfaction in 98.7% of cases versus 70.5% in non-pain freedom attacks. However, the largest gradients in satisfaction were observed for pain relief ≥50% (93.1% vs. 22.2%) and absence of rescue medication (92.7% vs. 25.7%), suggesting that satisfaction is driven primarily by completeness of relief and durability of effect rather than by partial pain reduction alone.

Adverse events at 2 h were reported in 64 of 320 treated attacks (20.0%) and by 30 of 111 patients (27.0%). The reported events were predominantly mild to moderate and consistent with the safety profiles of both sumatriptan and naproxen. The most frequent category was gastrointestinal (epigastralgia, dyspepsia, GERD episodes), reported in 22 attacks (6.9%) and 10 patients (9.0%), in line with the known gastrointestinal toxicity of NSAIDs. This was followed by dizziness in 12 attacks (3.8%) from 5 patients (4.5%), chest tightness or chest pressure in 11 attacks (3.4%) from 5 patients (4.5%), and somnolence/fatigue in 8 attacks (2.5%) from 4 patients (3.6%). At 24 h, AEs were reported in only 6 of 320 attacks, while all 24-h events represented persistence of 2-h events rather than new-onset late reactions (Table 3). No patients were recorded as discontinuing treatment due to adverse events or to lack of effectiveness.

**Table 3:** Description of safety and tolerability.

AE category	n (%) attacks	N (%) patients
Any AE	64 (20)	30 (27)
Epigastralgia/dyspepsia/GERD	22 (6.9)	10 (9)
Dizziness	12 (3.8)	5 (4.5)
Chest tightness/chest pressure	11 (3.4)	5 (4.5)
Somnolence/fatigue	8 (2.5)	4 (3.6)
Non-specific pain/discomfort	3 (0.9)	3 (2.7)
Paraesthesia	3 (0.9)	2 (1.8)
Nausea/malaise	3 (0.9)	2 (1.8)
Increased blood pressure	3 (0.9)	1 (0.9)
Neck pain	3 (0.9)	1 (0.9)
Dry mouth	1 (0.3)	1 (0.9)
Cognitive dysfunction (brain fog)	1 (0.3)	1 (0.9)

## Discussion

In this prospective Greek multicenter real-world study, fixed-dose sumatriptan 85 mg/naproxen sodium 500 mg was associated with 2-h pain freedom in 23.8% of treated attacks, 2-h pain relief in 68.8%, and sustained pain freedom in 21.9%. The most clinically informative findings were the high rates of full return to normal activities at 2 h and avoidance of rescue medication, observed in 64.4 and 76.9% of treated attacks, respectively. These findings suggest that in routine headache-center practice the combination may provide meaningful attack control even when strict 2-h pain freedom is achieved in only a minority of attacks.

In routine headache practice, functional recovery and avoidance of rescue medication may be more informative than strict 2-h pain-free rates alone, because they indicate whether an attack has been sufficiently controlled to allow patients to continue work, household tasks, social participation, and other daily living activities without additional medication intake. This particularly applies in real-world settings where patients often present with high baseline pain intensity, previous triptan exposure, chronic migraine, comorbidities, and variable timing of treatment (21–23).

Return to function is particularly important when compared with the pivotal randomized trials by Landy and colleagues. In those two double-blind, placebo-controlled phase 3 studies, sumatriptan/naproxen sodium 85/500 mg allowed significantly more patients to reach normal or mildly impaired functioning earlier than placebo, sumatriptan, or naproxen monotherapy, with higher functional rates already evident at 2 and 4 h. Participants treated a single moderate or severe attack under protocol conditions, and the functional scale distinguished normal function from mild impairment, severe impairment, or bed rest (18). Our study used a stricter real-world binary endpoint, counting only an explicit “yes” for full return to normal activity and classifying partial return as non-response. Despite this conservative definition and the absence of trial-level selection, nearly two-thirds of attacks in our cohort achieved full functional recovery by 2 h. This supports the view that the fixed-dose sumatriptan 85 mg/naproxen sodium 500 mg the combination’s value extends beyond analgesia to the restoration of everyday living activities (18).

Our findings are also consistent with the pivotal efficacy trials reported by Brandes and colleagues, wherein sumatriptan/naproxen sodium was superior to placebo and showed advantages over its individual components for several sustained outcomes, including lower rescue medication use and lower recurrence (11). A subsequently published literature review similarly emphasized that the combination improves loss of normal function and reduces rescue medication compared with placebo or monotherapy in controlled settings (24). Our no-rescue medication intake rate of 76.9% supports the established pharmacological rationale of the combination with the triptan component providing migraine-specific early control, while naproxen may add durability and help avoid attack recurrence through its longer anti-inflammatory effect. Importantly, only 6 of 76 attacks that were pain-free at 2 h in our study failed to remain sustained pain-free at 24 h, indicating that early complete responders rarely needed later escalation.

Another clinically important issue raised by our findings relates to the comparison with longer-term data. Smith and colleagues reported a 12-month phase 3 open-label multicenter study in which 565 participants treated 24,485 migraine attacks with sumatriptan/naproxen sodium. In that setting, 70% of attacks were treated with one dose and 81% achieved pain relief, while 60% were pain-free at 2 h, with sustained improvements in treatment satisfaction and migraine-specific quality of life metrics (25). Those rates are higher than ours for pain freedom, but methodological differences may account for discrepancies between results. Open-label continuation studies may retain patients who tolerate and benefit from treatment, and they do not reproduce the heterogeneity of routine specialist practice. By contrast, our cohort included a substantial CM subgroup, high baseline pain intensity, and extensive prior acute-medication exposure. Thus, our lower 2-h pain-free rate but high functional recovery and high no-rescue rate may better represent the expected effectiveness of the combination when used in everyday headache-center daily practice.

The repeated-attack data further support the effectiveness of the fixed-dose sumatriptan 85 mg/naproxen sodium 500 mg combination, because return to function increased from 55.9% in the first attack to 66.4 and 71.6% in the second and third attacks, while the no-rescue medication rate longitudinally increased from 73.9 to 77.6 and 79.4%. Although notable, this pattern warrants cautious interpretation, because treatment timing, patient selection across attacks, and within-patient correlation were not controlled. Nevertheless, it allows us to suggest that patients who continue using the combination may experience stable or improving therapeutic benefit across consecutive attacks. This is clinically relevant because the value of acute migraine treatment should be defined by recording the clinical outcome over repeated attacks, rather than after a single episode.

The early-treatment data may also help explain the functional recovery results. Treatment within the first hour was associated with markedly better full return to function than later treatment (71.2% vs. 45.2%, *p* < 0.001), and also higher pain relief (74.6% vs. 52.4%, *p* < 0.001). These findings are aligned with randomized early-intervention studies, where sumatriptan/naproxen sodium performed better when taken while pain was still mild and before sensitization was established (7). In our setting, early intake did not significantly increase 2-h pain freedom, probably because of limited power, high baseline severity, or confounding by attack characteristics. However, its clear association with clinically-relevant functional recovery suggests that educating patients on early acute medication use may be essential before escalation of pain intensity and sensitization (26); particularly when the therapeutic goal is functional recovery rather than pain reduction alone.

Resolution of associated symptoms was also observed, especially for nausea or vomiting, which resolved in nearly two-thirds of affected attacks. Photophobia and phonophobia resolved in approximately half of affected attacks. These data support the relevance of evaluating migraine as a phenotype rather than assessing head pain alone. However, the study did not prospectively collect a formal most-bothersome-symptom (MBS) endpoint, so these findings should be described as associated symptom outcomes rather than as formal MBS results. Nonetheless, the MBS improvement supports the view that the combination acted on the migraine attack as a syndrome, rather than only reducing head pain intensity (27).

Our subgroup findings are also of interest. Patients with EM had significantly better outcomes than those with CM for two-hour pain freedom, sustained pain freedom, return to function, and no rescue medication. This is because CM is associated with higher attack frequency, central sensitization, medication overuse risk, psychiatric comorbidity, and greater baseline disability, all of which may reduce apparent acute treatment success (28, 29). The absence of a significant difference by psychiatric comorbidity should be interpreted cautiously, because the subgroup analysis was underpowered, while psychiatric diagnoses were not phenotyped by severity or current treatment. Nonetheless, our findings demonstrate that psychiatric comorbidity did not appear to diminish treatment benefit in our dataset (30).

The tolerability profile was consistent with known triptan and NSAID effects. Adverse events were reported in 20.0% of attacks at 2 h and by 27.0% of patients. Gastrointestinal symptoms were the most common adverse-event category, followed by dizziness, chest pressure or tightness, and somnolence or fatigue. Chest symptoms and increased blood pressure require careful clinical interpretation in any triptan-treated population, even when events are transient and non-serious. The present study did not include structured cardiovascular assessment at the time of symptoms, formal event adjudication, or systematic severity grading. Accordingly, although the findings should be interpreted as real-world tolerability observations, rather than definitive safety estimates, they also reinforce the need for careful patient selection; particularly in those with gastrointestinal vulnerability, uncontrolled hypertension or cardiovascular disease (7, 19).

This study has several strengths. It was prospective, multicenter, and conducted under routine clinical conditions in Greek headache practice. It assessed up to three consecutive treated attacks and included outcomes that matter directly to patients and clinicians, including functional recovery, rescue medication use, sustained response, associated symptom resolution, treatment timing, migraine subtype, and tolerability. All these secondary outcomes directly inform clinical decision-making in acute migraine care.

The study also has important limitations. The single-arm observational design precludes causal inference and does not allow direct comparison with placebo, monotherapy, or alternative acute treatments. Repeated attacks from the same patient were not independent, and formal mixed-effects or generalized estimating equation models were not applied. The early-treatment analysis was unadjusted and should be interpreted as associative. The female preponderance (80.2%) in our cohort might also be perceived as a limitation, although this gender distribution is entirely consistent with the well-established epidemiology of migraine, which is approximately three times more prevalent in women than in men, particularly during reproductive years (1, 2). Furthermore, subgroup analyses were limited by modest sample size, particularly for CM and psychiatric comorbidity. Subsequently these subgroup analyses should be interpreted cautiously. Satisfaction, treatment preference, medication-overuse status, preventive treatment use, and formal most-bothersome-symptom endpoints were not captured. Adverse events were patient-reported through a binary field and a free-text description; they were not systematically coded against a structured MedDRA dictionary, severity grading was not captured, and ancillary investigations (e.g., ECG or blood pressure measurement at the time of chest tightness or hypertensive symptoms) were not included. Causality assessment was therefore not possible and AE rates should be interpreted as crude incidence proportions.

In conclusion, this prospective Greek real-world study suggests that fixed-dose sumatriptan/naproxen sodium is an effective acute treatment for migraine in headache practice, particularly for pain relief, full return to normal activities, and avoidance of rescue medication. The strict 2-h pain-free rate was modest, but functional outcomes were favorable. Better outcomes were observed among patients with episodic migraine and in attacks treated within 1 h, although these findings require cautious interpretation because of the observational design. Tolerability was consistent with the expected profiles of sumatriptan and naproxen. Careful patient selection remains essential, particularly in individuals with gastrointestinal vulnerability, uncontrolled hypertension, cardiovascular disease, renal disease, or other NSAID or triptan-related risk factors.

## Data Availability

The original contributions presented in the study are included in the article/supplementary material, further inquiries can be directed to the corresponding author.
